# Commentatio Medico-Practica de Morbis Intestini Cæci, et de dignitate hujus Visceris Pathologica in dijudicanda Passione Colica et Iliaca

**Published:** 1836-04

**Authors:** 


Art. IV.—*Commentatio Medico-Practica de Morbis Intestini Cæci, et de dignitate hujus Visceris Pathologica in dijudicanda Passione Colica et Iliaca.* Auctore Lud. Herm. Unger, Med. et Chir. Doct. &c.—*Lipsiæ*, 1828. 8vo. pp. 69.

*On Phlegmonous Tumours in the Right Iliac Region.* By J. M. Ferrall, Esq., Member of the Royal College of Surgeons in Ireland, Surgeon to the Maison de Santé and the Institution for the Treatment of Scrofulous Complaints.—*Edinburgh*, 1831. 8vo. pp. 24.

*Observations upon a peculiar Disease of the Cæcum, or Caput Coli; contained in a Letter to* Dr. Crampton, *from* Francis W. Smith, m.d., one of the Physicians in Ordinary to his Excellency the Lord Lieutenant; Fellow of the College of Physicians (in Ireland), &c. &c. &c.—Dublin, 1835. 12mo. pp. 47.

We have no desire to conceal the fact that it was the appearance on our table, among other new publications, of the work of which the title stands last in the above list, which made us refer to our shelves for the two which precede it, and which, it will be observed, are of considerably older date; and, as it was the nature of the contents of the more recent work which induced us to recur to the others, we shall, in the following remarks, give it our chief attention, and refer to them only for incidental illustrations of the subjects we may have to consider.

In noticing the little work of Dr. Smith, it is impossible to be honest without finding fault; and if in so doing we should offend the author, he may rest assured that it is a sense of duty only and the consciousness of the high responsibility of our office, and no pleasure in finding fault, that guides our pen. And, indeed, if ever any man was bound to put up patiently with the critic's award, whatever that might be, surely Dr. Francis Smith is the man, for nothing can be more evident than that his appearance as an author—at least as the author of a separate work—is perfectly uncalled for. Had, indeed, the scanty series of observations contained in this diminutive volume appeared as a communication to a medical journal, or had they obtained a place in the Transactions of the respectable Association* before the members of which they appear to have been originally read, they might have escaped without censure, if they had obtained no commendation. But when they come forth to the public in the shape they now possess, as a distinct volume, brief indeed, yet formally divided into chapters (actually twelve chapters in forty pages!) and with no slight pretensions on the part of the author to originality of views and improvements in practice, it is not possible to overlook them, more especially as they relate to a subject of high pathological interest and practical importance.

It is now many years since our attention was first called to the particular disease noticed in the present pamphlet, by the occurrence of several cases in our own practice; and while we were hesitating whether we should make them the subject of a brief communication to some of our journals, we were fortunately anticipated, by the notice of the same affection by some of the most distinguished French pathologists, who gave to the profession a much fuller account of it than our limited experience could have supplied; and, as they took the same view of it which had occurred to ourselves, we had nothing to regret, but every ground for satisfaction, that the subject had fallen into other hands.†

Nearly about the same time the little work, the first on our list, appeared at Leipsig, although we were not aware of its existence till some time afterwards. In it the author gives a complete but brief view of all the principal affections to which the cæcum is liable, and notices in a particular manner its local inflammatory states, less fully indeed than the French authors, but infinitely more so than Dr. Francis Smith.

Some years after this, the very excellent paper on the same subject, the second on our list, was published by Mr. Ferrall, of Dublin, first in the Edinburgh Journal for July 1831; afterwards separately. The substance of this we are told by its author, "was prepared before the Memoir from M. Dupuytren's *Clinique* appeared," and some of the cases in which bear date as far back as the year 1823. We are thus particular in referring to dates, because Dr. Smith, in the pamphlet before us, the whole of which is devoted to the consideration of the early stages of the same

* Association of Physicians in Dublin.

† See a Memoir on Phlegmonous Tumours of the right iliac fossa (from the Clinical Lectures of Dupuytren), by P. Méniere, M.D., Archiv. Gén. t. xvii., republished in Dr. Johnson's Med. Chir. Rev. Nov. 1828.

identical affection, has thought proper to represent it as having "met with the most extraordinary neglect on the part of medical authors," (p. 8,) and of having been "most unaccountably doomed to comparative obscurity." (Introd. p. vi.) If one were, indeed, to judge of the acquaintance of the members of the profession, generally, with this affection, from that which seems to be possessed by this author, we should certainly regard it as most limited, seeing that he does not once refer to the French authorities, and never alludes to Mr. Ferrall's Memoir, until towards the close of his own essay, when he refers to it for a description of some "cases of the disease in a more aggravated and lethal form." (P. 37.) It is singular, also, that, with the account of the disease by the French authors, to which we have referred as existing in the pages of an English journal in every body's hands, he should have added, in reference to this Memoir of Mr. Ferrall, "there is not, I believe, in our language, any other printed account of the disease."(P. 37.) And it is, certainly, equally singular, that acquainted as he here acknowledges himself to be with Mr. Ferrall's paper, he should have preserved an utter silence respecting it while discussing the pathology, diagnosis, and treatment of the complaint, all of which points Mr. Ferrall discusses, and should have only incidentally noticed it when about to close his work, by the detail of his own few trifling cases. Dr. Smith may, possibly, shelter himself under the plea, that his observations apply, and were only intended to apply, to the incipient or chronic form of the disease described by the French writers and Mr. Ferrall; but, admitting that such is the object of his Essay, we hold him not the less bound to have made himself acquainted with their writings and opinions, before he put himself forward as a teacher of others. And even this excuse will not serve in the case of Dr. Unger, since his remarks apply both to the chronic and acute form of the disease, and indeed do not include the extreme forms of it described by Dupuytren and Mr. Ferrall. It is hardly a satisfactory excuse for Dr. F. Smith's ignorance of Dr. Unger's book, that it might be known only to few persons in this country. He might have become acquainted with it without any difficulty, as it was announced in all the foreign catalogues for some years, and as it was written in a language with which all physicians are presumed to be acquainted. At least, we think it would be prudent in writers to take some pains to acquire a knowledge of the literary history of the subjects on which they write, before they publish their writings; more particularly if they make any pretensions to novelty.

After all that has been said, and although we may have yet further fault to find with Dr. F. Smith on other grounds, we believe that his little pamphlet will be of use, by calling anew the attention of the younger members of the profession to this interesting form of abdominal disease, and by soliciting the notice of all to its earlier stages. Regarded as a chapter in the more important memoirs of MM. Dupuytren, Unger, Husson, Dance, Méniere, and Ferrall, it is not without its value; and had the author been content with claiming for his observations the meed to which they are entitled, and extended a common measure of justice to his fellow-labourers in the same field of enquiry, he would have received from us nothing but commendation.

For the benefit of such of our readers as may not be acquainted with the memoirs referred to, it may be useful here to state, that the affection under consideration, and which we think very improperly designated by M. Dupuytren and Mr. Ferrall by the name of "Phlegmonous Tumour," is a local disease of the cæcum, and although, perhaps, not in all cases essentially and primarily inflammatory, yet hardly ever attracting the attention of the physician, or even of the patient, until exhibiting evident signs of phlogosis of that bowel, or, still more commonly, of that and the tissues exterior to it and immediately surrounding it. "Herbam crevisse apparet," says Dr. Unger, "non apparet crescere." (P. 38.) In certain cases, (and Mr. Ferrall's first case appears to have been of this kind,) the local affection seems to consist simply of a painful over-distension of the bowel from fæcal accumulation, arising from some of the various causes of disease to which this portion of the intestinal canal is, from its peculiar functions, liable. Dr. Unger considers fæcal accumulation, from neglect in yielding to the natural stimulus to empty the bowels, as the most common cause. (P. 42.) In general, however, the disease is of an inflammatory character from the beginning, consisting essentially of a slight chronic inflammation of the mucous membrane of the *caput cæcum,* producing a stoppage of its peristaltic action, and consequently an accumulation of its contents, with perceptible tumour of the part, and, eventually, the extension of the inflammation to the exterior of the bowel, with all the ordinary accompaniments of a local and superficial phlegmonous inflammation superadded to the internal intestinal disease. The cause of this peculiarity of the cæcal inflammation extending to the adjoining tissues is found in the fixedness of this bowel, and in the peculiar functions it has to perform, and the consequent disarrangements to which it is liable. This inflammatory affection of the cæcum may be divided into three stages or degrees, first, when the inflammation is slight, and almost or altogether confined to the coats of the intestine; secondly, when it involves the surrounding parts, and presents the characters of a local phlegmon, seated more or less deeply; and thirdly, when suppuration has supervened in the cellular tissue, and the escape of the pus by the surface, or by perforation of the bowel, has produced a disease of a complex character and of a very dangerous nature. Of these three forms of the disease, the first and second are, fortunately, by much the most common; and the second is probably that which is most commonly met with in practice, owing to the preliminary stage being often overlooked, or not sufficiently attended to, to be the subject of the physician's or surgeon's care. The last is extremely rare.

The only part of Dr. Smith's account of the disease that possesses the least interest, is his description of the early stage; but this is so overloaded with minutiæ which have no essential connexion with the particular pathological state in question, that no distinct picture is left on the reader's mind. Positive and negative symptoms are jumbled together in the strangest confusion; many are noticed as present which have nothing to do with the disease, and many are recorded as absent which the disease can never have any thing to do with. We therefore prefer to extract a brief account of it from the work of Dr. Unger.

"The invasion of this affection is very insidious, as its early progress is accompanied by little inconvenience, hardly calls the attention of the patient to it, or, at least, is not deemed deserving of medical aid. Step by step however it advances, silently undermines the health, and finally gives rise to imminent danger. No single symptom is of sufficient weight or constancy to indicate the presence of this affection: all must be viewed in connexion, and carefully, in order to establish the diagnosis. In the more simple cases the following are the ordinary marks of the disease: the patient does not lose his strength, but abates somewhat in his wonted activity, and he looks ill. Pains in the iliac and colic regions, somewhat of a periodical character, go and come; sometimes they extend over the whole abdomen, and are felt most during the period of digestion, when the peristaltic action is at its height. There are few signs of any gastric affection, except a reddish tongue and some thirst; and the appetite is not quite gone. The patient becomes low-spirited, seeks for ease on the couch, where he prefers to lie on the right side with the thighs drawn up; he soon wearies, gets up discontented, but shortly lies down again. The pulse is small. The alvine excretions are unhealthy; the bowels are at one time confined, at another relaxed, the stools being neither large nor figured, but loose and mucous, and passed without producing the expected alleviation of the previous pains. If at this stage we examine the abdomen, we find it in general of the natural form, soft and not tender as in the case of ordinary inflammation, but if we press forcibly in the right iliac region so as to depress the integuments to a considerable depth, we produce a feeling of pain." (P. 41.)

The following is an extract from Dr. Smith's work, descriptive of the same stage of the disease, purged, however, (a liberty which we hope the author will excuse,) from not a little of the extraneous matter and repetitions by which the original is disfigured. This extract will, we hope, satisfy our readers that the English author has less cause than he imagines for pluming himself on his originality.

"The first symptoms complained of by the patient are, dull pain of the right side, which can be increased by pressure; an inability, or, more properly speaking, a disinclination to go to sleep on the left side; the bowels present a great degree of irregularity in their action, the stools being for the most part dry, scanty, and unfrequent, and not unusually clayey in colour, and imperfect in consistency; slight diarrhœa, generally appearing from time to time at intervals of uncertain duration; the urine is in most cases high-coloured, and abounding in animal salts; occasionally, however, it is natural in its appearance and qualities. The appetite, generally, is undisturbed; the tongue sometimes slightly furred, and of a whitish or yellowish colour in the mornings, but is as frequently of a healthy colour and appearance; the pulse is slower and more languid than in health; the countenance begins to assume an unhealthy, yellowish appearance; the individual loses flesh. As the disease advances the pain becomes more distressing, and the region in which it makes itself felt more extensive; from having at first been felt as a dull, undefined pain in the right side, the patient now feels it in the whole region lying between the cartilages of the false ribs on the right side, and the crest and spine of the ilium. The stomach still continues to perform its functions, but nutrition does not take place in a sufficient degree to repair the waste of the body; the liver begins to become irregular in its action; flatulency of the bowels becomes a distressing symptom. The skin now acquires a preternatural dryness, and the patient altogether acquires a sunk and marasmatic appearance. If at this period we subject the patient to a suitable examination, we shall find that, if we make pressure downwards and to the right side, we shall very considerably increase the sufferings of the patient; and, if we commence our pressure from a point midway between the navel and the superior anterior spinous process of the ilium, and carry it in a direction downwards, outwards, and to the right side, we shall soon arrive at a point where the pain reaches its maximum. The pain is frequently described as shooting and radiating in all directions from under the finger, but principally in an upward direction. I have in a few instances known the pain to extend, under those circumstances, even up to the point of the shoulder; we shall, at the same time, in general be able to discover a tumour of a pretty considerable size, hard consistency, and irregular figure, occupying and swelling upwards from the iliac fossa." (P. 12.)

The only defective point in the description of Dr. Unger, is his omission of the *tumour* as well as tenderness in the right iliac region. Indeed this author, although possessing a far superior knowledge of the disease to Dr. Smith, appears still to have had only a very imperfect acquaintance with it, especially in its latter stages. For an account of these we must, therefore, refer our readers to the excellent paper of Mr. Ferrall, in the Edinburgh Journal, and that of M. Méniere in the Medico-Chirurgical Journal; and we must add, that it is to these also, and not the pamphlet of Dr. Smith, that they must look for rational views of pathology and grounds of diagnosis, although the last-named writer has bestowed a whole chapter on the latter point.

Too much, however, even as to physical extent of matter, must not be supposed to be implied, in the case of Dr. Smith's book, by the title of *chapter*, as the reader will be ready to allow on perusing the one (Chap. iv.) which we shall here transcribe *entire;* partly on this account, and partly because we do not quite agree with Dr. Smith as to the statements contained even in it.

"Chap. iv. To the disease, as I have described it, I should be disposed to assign the name of *cæconitis;* and as such a term would be in strict accordance with the rules which guided those who have bestowed names on the morbid affections of the other portions of the intestinal canal, I do not foresee any objection to its being adopted." P. 25.

Now we rather think that Dr. Smith may find persons disposed to object to this term, and even to question whether it is "in strict accordance with the rules" to which he refers. We, for our parts at least, are altogether ignorant of there being such a word as *cæcon;* but, even if it were justifiable, for the sake of euphony, to read *cæconitis* in place of *cæcumitis* (and we really see no great difference between them,) Dr. Smith ought to know that the rules which should guide the formation of such terms—broken, occasionally, we admit—forbid the addition of the Greek particle *itis* to a strictly *Latin* word like *cæcum.*

The treatment of this complaint, in the early stage of it under consideration, is sufficiently obvious. The principal indications are to subdue the inflammation by the local application of leeches and fomentations, and to remove the accumulation of fæces by the most effectual yet mildest means. After the removal of the pain, Dr. Smith's favourite practice is to give a well-known pill, consisting of a grain and half of aloes, rubbed up with gum mastick, ipecacuan and soda, twice a day, combined with a mild bitter infusion; and the practice is very judicious, so long as there is no inflammatory state present, and the pills have the effect of keeping up a moderate and steady action on the large intestine.

"In general (says Dr. S.) a few weeks of well directed care will free the individual from the disease; and it will then be only requisite, that by an occasional dose of the pills he should guard against allowing the cæcum to again become over-distended. Occasionally, however, [and we ought to apprize the reader that the last sentence terminated Chap. vi. while this begins Chap. vii.] we meet with cases which do not so easily yield, even to the most admirably directed treatment.—In these cases, *I am in the habit of making use of an invention of my own,* which has the effect of keeping up the evacuations from the bowels, and stimulating powerfully, and in a healthy and tonic manner, all the abdominal viscera, including, of course [*of course*], the cæcum and colon." P. 29.

The invention is thus announced:—

"I am in the habit of ordering the patient, on rising in the morning from bed, to have a *douche of cold water* of about half an inch in diameter, and of a very moderate force, (never exceeding a pound or a pound and a half at the commencement,) to be made to play for a period, varying from a few seconds to a couple of minutes, upon the abdomen, and to let the stream be principally directed towards the seat of the disease. Even common dashing of the abdomen from a large sponge, or a stream directed from a moderate height, by means of a watering-pot, divested of its rose, may be made to answer; but a douche from a leathern *hydraulic* tube, having a height of water of three or four feet to supply the force, and a diameter of half an inch, is the most certain and complete manner in which it can be applied.—Now the effects of this affusion are very remarkable; the bowels are stimulated to action in a very decided and very satisfactory manner.—If made use of as the patient rises from bed, by the time the toilette and breakfast have been gone through, the bowels will be ready to act with certainty and with success, and the stools will be both copious and of a satisfactory consistency." P. 31.

How far this practice is altogether an invention of Dr. Smith's,—how far it is likely to be admissible in many cases,—what proportion of patients are likely to submit to it,—and whether the practical consequences are fairly and logically deduced from the premises, we have not time to enquire; as we have already bestowed more space on this pamphlet than we can well spare. We may, however, just suggest a doubt whether two subsequent statements made by the author, viz. that he "*always associates* with its use the soothing influence of the warm bath," (P. 32,) and that "after some time, it will *in most cases be found requisite* to return to the use of the aloetic medicines," (P. 33,) may not be supposed by some to weaken the force, if not the legitimacy, of his deductions.

In his CHAP. XII. the author says that "some general statistical observations have suggested themselves to his mind, but that he prefers passing that part of the subject over in silence; only remarking that individuals of all ages between twenty and sixty are alike liable to the disease." "That it occasionally proves fatal *is quite certain;* the daughter of an eminent legal character in this city [Dublin], as also the child of a French nobleman, both, to my knowledge, succumbed under incurable cæconitis." (P. 46.) When it is observed that several of the very cases referred to by Dr. Smith himself, in Mr. Ferrall's paper, were *fatal* cases, the *possibility* or certainty of such an event seemed hardly to require to be established by the cases of the children of the eminent and noble personages 'within his own knowledge.'

In addition to what may be called the technical or medical blemishes in Dr. Smith's work, we cannot lay down our pen without entering our protest against his avowed design of writing it for *the public,* as well as the members of the profession. "I have been at length induced (he says) to give to the public, *medical and otherwise,* the fruits of my experience." "Pathological details (he says in another place) I have as much as possible avoided, as they would be wholly unintelligible to the popular reader." (P. 46.) Now, we ask, what have the non-professional public to do with such a subject as that which forms the whole of Dr. Smith's work? One more strictly medical and professional, it would not be easy to point out; and when we see these avowed appeals to those who cannot profit by its details, coupled with the very unusual and minute mode of designating the author's locality, in the Introduction, we cannot but doubt whether, in committing his observations to the press, he was not partly influenced by motives which, in the ancient days of physic at least, would have been deemed hardly becoming in a member of its highest order.

